# Establishment and Characterization of a Brain Parenchymal Metastatic Cell Line AlmoR1 Derived From an NSCLC Patient With EGFR‐TKI Resistance

**DOI:** 10.1002/cam4.70827

**Published:** 2025-04-02

**Authors:** Jingyi Wu, Xiangfei Wu, Jing Wang, Guili Feng, Yuhan Wang, Zide Chen, Wei Wang, Rong Wang

**Affiliations:** ^1^ Department of Radiation Oncology, Nanfang Hospital Southern Medical University Guangzhou Guangdong People's Republic of China; ^2^ Department of Neurosurgery Guangdong Sanjiu Brain Hospital Guangzhou Guangdong People's Republic of China; ^3^ Department of Interventional Radiology, Guangdong Provincial People's Hospital (Guangdong Academy of Medical Sciences) Southern Medical University Guangzhou Guangdong People's Republic of China

**Keywords:** AlmoR1, brain metastasis, NSCLC, primary culture, the resistance of third‐generation EGFR‐TKIs

## Abstract

**Background:**

Non‐small cell lung cancer patients with EGFR mutation have a high rate of brain metastases, and EGFR tyrosine kinase inhibitors (TKIs) are the principal therapeutic approach. However, acquired targeted therapy resistance is the main reason for EGFR‐TKIs' treatment failure. At present, the mechanism of intracranial acquired targeted therapy resistance is limited, mainly due to the lack of a cell line that can be used for its study.

**Methods:**

A brain parenchymal metastatic sample that progressed after third‐generation EGFR‐TKI treatment was used to establish a cell line named AlmoR1. The genetic characteristics of the cell line were evaluated by short tandem repeat (STR) profiling and whole‐exome sequencing analysis. The phenotypic characteristics were characterized by CCK8, western blot, HE staining, immunohistochemistry (IHC), and orthotopic brain tumor model.

**Results:**

The cell line we successfully established, AlmoR1, can be passed in vitro stably. STR analysis revealed it was a novel NSCLC BM cell line. It harbors the EGFR E746_A750del (ex19del) mutation, and the IC50 to almonertinib and osimertinib of AlmoR1 was significantly higher than that of sensitive cells. In our orthotopic brain tumor model construction with AlmoR1 cells, 75% (3/4) tumor formation can be observed in the living system.

**Conclusions:**

These data suggest that the established cell line, AlmoR1, preserved the resistance to broad third‐generation EGFR‐TKIs and good tumorigenicity in an intracranial orthotopic model, so that it can serve as a new tool to elucidate the pathogenesis, explore new treatment methods, and conduct the development of new drugs for targeted therapy resistance of brain metastases.

## 1 Introduction

Non‐small cell lung cancer (NSCLC) accounts for approximately 80%–85% of all lung cancer cases and is one of the leading causes of cancer‐related deaths worldwide [1, 2]. Brain metastasis (BM) is a critical factor influencing the prognosis of NSCLC patients, with an estimated 20%–40% of NSCLC patients developing brain metastases [3, 4]. Epidermal growth factor receptor (EGFR) is the most common driver gene in lung cancer, and its mutation is associated with an increased incidence of BM [5, 6]. Studies have shown that the cumulative incidence of BM in advanced NSCLC patients with EGFR mutations increases over time, with a 5‐year incidence rate reaching as high as 53% [7].

Currently, EGFR tyrosine kinase inhibitors (TKIs) are widely used in the treatment of NSCLC patients with EGFR mutations, spanning across various stages, including first‐line treatment for advanced NSCLC and both neoadjuvant and adjuvant therapies for early or locally advanced NSCLC [8, 9, 10]. In particular, compared with conventional regimens, third‐generation EGFR‐TKIs (e.g., osimertinib, almonertinib) have significantly extended median survival time in patients with BM from 2–3 months to 10–19 months [11, 12, 13, 14, 15]. However, acquired resistance remains the primary cause of treatment failure with targeted therapies, and treatment options for intracranial resistance are still extremely limited [16, 17]. Thus, there is an urgent need to explore the mechanism of intracranial targeted therapy resistance.

Nevertheless, due to the unique intracranial microenvironment and the challenges of obtaining BM samples, current research on resistance mechanisms has largely focused on extracranial lesions, with a lack of studies addressing intracranial resistance to targeted therapies. The limited existing research on intracranial resistance mechanisms has mainly focused on leptomeningeal metastases, given that cerebrospinal fluid (CSF) samples are easier to obtain [18, 19]. Studies on targeted therapy resistance in brain parenchymal metastases remain scarce. Meanwhile, current studies on brain parenchymal metastases primarily focus on their occurrence, with little exploration into why they develop resistance [20, 21]. This is largely due to the limited availability of NSCLC brain parenchymal metastatic cell lines, especially those with driver gene mutations and resistance to third‐generation EGFR‐TKIs.

In this study, we successfully established a human‐derived intracranial targeted therapy resistant cell line, AlmoR1, from a brain parenchymal metastatic sample that progressed after 14 months of third‐generation EGFR‐TKI treatment. Whole‐exome sequencing (WES) confirmed that the cell line harbors the EGFR E746_A750del (ex19del) mutation and exhibits broad resistance to EGFR‐TKIs in vitro. Additionally, the AlmoR1 cell line demonstrated good tumorigenicity in an intracranial orthotopic model. This novel cell line not only fills a gap in the current research tools but also provides a unique platform for studying the resistance mechanisms of NSCLC brain metastases and facilitating the development of novel therapeutic strategies.

## 2 Methods and Material

### 2.1 Patient's Clinical Information

The clinical specimen was taken from a 72‐year‐old non‐smoking Chinese female patient diagnosed as NSCLC with BM. The patient underwent 14 months of TKI treatment and triple resection of brain metastases. This study was approved by the Ethics Committee of Guangdong Sanjiu Brain Hospital (Affiliated Brain Hospital of Jinan University) (Ethical No. 2024‐02‐064), and written informed consent was obtained from the patient prior to the procedure.

### 2.2 Primary Cell Extraction and Purification

A fresh BM specimen was obtained from an NSCLC patient undergoing a third BM resection, immediately preserved in tissue preservation solution (#DWX1001, Dowobio, China), and transported at 0°C to a biosafety cabinet. The tissue was rinsed three times with PBS (#PB180327, Procell, China) containing 1% penicillin and streptomycin (#C100C5, NCM Biotech, China), minced using micro‐scissors (#XGJ1400, Stronger, China), and cultured overnight. The next day, the cell suspension was filtered through a 100‐μm cell strainer (#15‐1100, Biologix, China), followed by centrifugation at 300g for 3 min at room temperature (RT), and the primary cells were cultured in a humidified 37°C CO_2_ incubator using Roswell Park Memorial Institute (RPMI) 1640 (#PM150110, Procell, China) medium supplemented with 10% fetal bovine serum (FBS, #10270‐106, Gibco, US). Primary tumor cells were further purified using a differential adhesion method, where the cell suspension was cultured for 4 h and nonadherent cells were collected for subsequent culture. This process was repeated multiple times to selectively enrich tumor cells and minimize contamination from nontumor cells.

### 2.3 Short Tandem Repeat (STR) Profiling

Genomic DNA was extracted from the cultured cells (passage 60) for STR DNA profiling at GuangZhou Jennio Biotech Co. Ltd. (Guangzhou, China). The bioinformatics workflow was performed according to the procedures established at GuangZhou Jennio Biotech Co. Ltd. (https://en.jennio‐bio.com/). Briefly, genomic DNA was amplified by polymerase chain reaction (PCR) and assayed using an ABI 3730XL DNA Analyzer to determine the STR loci. The STR profiles were then compared with the profiles in public cell banks, including ATCC, DSMZ, JCRB, and RIKEN, for reference. Eight STR loci (CSF1PO, D13S317, D16S539, D5S818, D7S820, TH01, TPOX, and vWA) were matched for DNA fingerprinting analysis.

### 2.4 WES Analysis

WES was performed by BGI Genomics Co. Ltd. (Shenzhen, China). The AlmoR1 cells cultured for 7 days were sent to BGI Genomics Co. Ltd. to perform the WES by DNBSEQ. Primary analysis was performed by base calling. The raw data of exome sequencing were analyzed by bioinformatics analysts of BGI Inc. The GATK4 HaplotypeCaller was used to call SNV and InDel. The variants were annotated by Ensemble‐VEP. The results were analyzed using R (R Core Team, 2023) and figures were produced using the package ggplot2 (Wickham, 2023).

### 2.5 Growth Curve

Cell proliferation was assessed using the Cell Counting Kit‐8 (CCK‐8; #C0005, TargetMol, US), following standard protocols across three independent replicates. Briefly, cells were plated in 96‐well plates at a density of 2 × 10^3^ cells per well and cultured under standard conditions (37°C, 5% CO_2_) for 7 days. At the designated time points, 20 μL of CCK‐8 reagent was added to each well and incubated for 3 h. The resulting formazan product was measured by recording the absorbance at 450 nm. Growth curves were generated based on the absorbance values to quantify cell proliferation.

### 2.6 Cell Viability Assay

Cell viability was evaluated using the MTT assay, as previously described [22]. PC9 and AlmoR1 cells were plated in 96‐well plates at a density of 2 × 10^3^ cells per well in 100 μL of RPMI‐1640 plus 10% FBS and incubated for 24 h. Cells were then treated with different concentrations (0, 0.01, 0.03, 0.1, 0.3, 1, 3, and 10 μM) of almonertinib (#HS‐10296, Selleck Chemicals, US) or osimertinib (#AZD9291, Selleck Chemicals, US) and incubated for 72 h. Following treatment, 50 μL of MTT solution (2 mg/mL; #E‐CK‐A341, Elabscience, China) was added to each well and incubated for 4 h at 37°C. The supernatant was removed, and the dark‐blue crystals were dissolved by adding 100 μL of dimethyl sulfoxide (DMSO). Absorbance was measured with an MTP‐120 microplate reader (Corona Electric, Corona, New York, US) at test and reference wavelengths of 490 and 550 nm, respectively.

### 2.7 Western Blot

PC9 and AlmoR1 cells were treated with 1 μM osimertinib or almonertinib for 72 h, washed with PBS, and lysed in RIPA buffer (#P0013B, Beyotime, China) containing protease and phosphatase inhibitors (Beyotime, China). Protein concentrations were measured with a BCA Assay Kit (#FD2001, Fudebio‐tech, China). Equal amounts of protein (20 μg) were separated by SDS‐PAGE (#0411/20528, Fdbio science, China) and transferred to polyvinylidene difluoride membranes (#IPVH00010, Millipore, US). Membranes were incubated overnight at 4°C with primary antibodies and then with secondary antibodies for 2 h at RT. Bands were visualized using an ECL Kit (Thermo Fisher Scientific). All experiments were repeated at least three times. The p‐EGFR (#2234), total‐EGFR (#2232), p‐AKT (#4060), total‐AKT (#9272), p‐ERK1/2 (#4376), and total‐ERK1/2 (#4695) antibodies used in the process were all purchased from Cell Signaling Technology. The internal reference β‐actin (#FD0060) antibody was purchased from FDBIO. The secondary antibodies used were M21001 and M21002 from Abmart.

### 2.8 Cell Cycle Analysis

The cell cycle was assessed using the cell cycle detection kit (KeyGEN BioTECH, China). PC9 and AlmoR1 cells were collected and plated in six‐well plates at a density of 2 × 10^5^ cells per well for 24 h, followed by adding 1 μM osimertinib or 1 μM almonertinib. After 72 h of incubation, the cells were collected, washed with PBS, and fixed with 70% cold ethanol at 4°C overnight. Following centrifugation (1000g, 3 min) and washing with PBS, 500 μL PI/RNase A staining solution (PI/RNase A ratio, 1:9; prepared in advance) was added, followed by incubation at RT in the dark for 30–60 min. Subsequently, the cell cycle was assessed using a BD FACScan flow cytometer. The results were analyzed using ModFit LT 4.0 (Verity Software House Inc.).

### 2.9 Establishment of an Orthotopic Brain Tumor Xenograft Model

The lentiviral vector Ubi‐MCS‐frefly_Luciferase‐3FLAG‐CBh‐gcGFP‐IRES‐puromycin was purchased from Genechem (Shanghai, China). AlmoR1 cells were transduced with lentiviral particles at a multiplicity of infection (MOI) of 30 in the presence of 8 μg/mL polybrene, and stable cell lines were selected using 2 μg/mL puromycin. Four‐week‐old female BALB/C nude mice (Guangdong Zhiyuan Biomedical Technology Co. Ltd., Guangzhou, China) were used for in vivo experiments. For the BM model [23], a cell suspension containing 3 × 10^5^ cells in 3 μL was stereotactically injected into the right striatum, 3 mm below the cortical surface, using a 10 μL Hamilton syringe equipped with a 26‐G needle. All animal experiments were conducted in accordance with the ethical guidelines of the Animal Experimentation Committee of Nanfang Hospital (Guangzhou, China) (approval no. IACUC‐LAC‐20230519‐001). Approximately 3 weeks postinoculation, tumor growth was monitored using noninvasive optical imaging to detect luciferase activity, as previously described [24], utilizing the Bruker In Vivo FX PRO Imaging System.

### 2.10 Immunofluorescence (IF) Staining

Third‐generation purified primary cells were selected for IF staining. IF staining was performed as previously reported [25]. Briefly, the tissues were fixed by 4% paraformaldehyde universal tissue fixative (#BL539A, Biosharp, China), embedded in paraffin, and then cut into 4‐μm sections. Then, the sections were incubated with pan‐CK (AB7753, Abcam, USA, dilution 1:200) conjugated with Alexa Fluor 488 (Thermo Fisher) and imaged under a fluorescent microscope green channel.

### 2.11 Immunohistochemistry (IHC) and Hematoxylin–Eosin (HE) Staining

Patient and xenograft tissues were immersed in 4% formalin and then sent to the Department of Pathology at Guangdong Sanjiu Brain Hospital for IHC and HE staining. The staining procedures were performed according to the protocols established by Guangdong Sanjiu Brain Hospital (Affiliated Brain Hospital of Jinan University). Ki‐67 (#GM027), CK (#GM302), TTF‐1 (#SPT24), and pan‐CK (#GM351529) antibodies were purchased from Gene Technology (Shanghai) Co. Ltd. Napsin A (#OT18A5) was purchased from Beijing Zhongshan Jinqiao Company. The dilution ratio was 1:100.

### 2.12 Statistical Analysis

Xenograft tumor progression data are presented as mean ± standard error (SE), while data from other experiments are shown as mean ± standard deviation (SD). Comparisons between two groups were conducted using Student's t‐test. All statistical analyses were performed using GraphPad Prism, Version 9.0 (GraphPad Software Inc., San Diego, CA, USA), with a two‐sided *p*‐value < 0.05 considered statistically significant.

## 3 Results

### 3.1 Acquisition of Third‐Generation EGFR‐TKI–Resistant NSCLC BM Specimens

**FIGURE 1:**
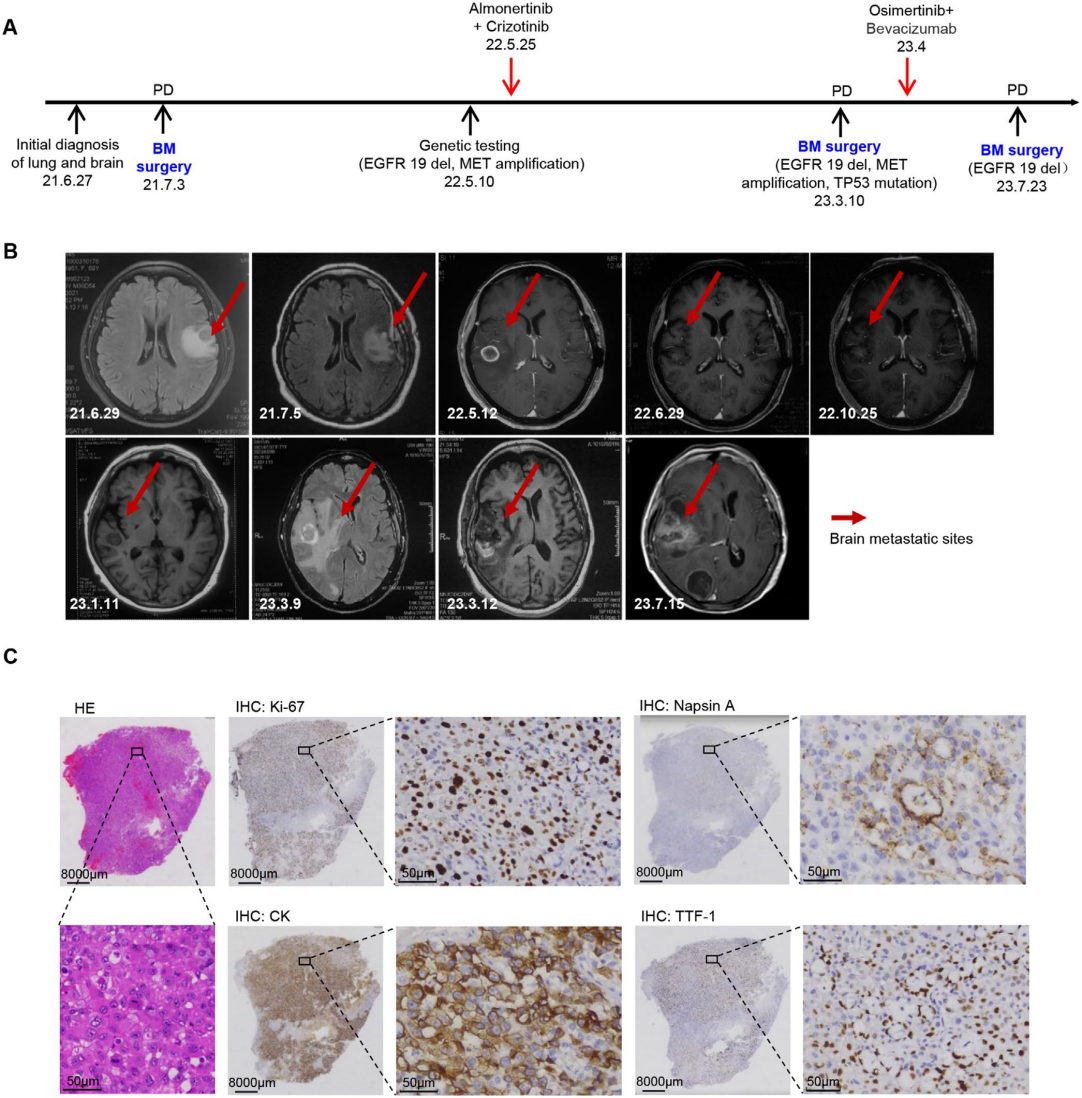
Acquisition of third‐generation EGFR‐TKI–resistant NSCLC brain metastasis specimens. (A) Timeline of the diagnosis and treatment of a case with EGFR mutation and resistance to third‐generation EGFR‐TKIs in a patient with NSCLC brain metastasis. (B) Corresponding to (A), the cranial magnetic resonance images of the patient at different stages of treatment. The brain metastases are indicated by the red arrows. (C) Hematoxylin and eosin (HE) and immunohistochemical (IHC) staining images of brain metastasis specimens from the last surgery (July 23, 2023), including Ki‐67, CK, Napsin A, and TTF‐1.

We obtained a BM tissue from an NSCLC patient harboring an EGFR mutation and resistant to the third‐generation EGFR‐TKIs. As shown in Figure 1A, this patient was diagnosed with BM of lung cancer in June 2021 and underwent BM resection in July. A follow‐up MRI in May 2022 revealed a new lesion in the right cerebral hemisphere, and pathology indicated an EGFR exon 19 deletion combined with MET amplification. Since May 25, 2022, the patient underwent treatment with a combination of almonertinib and crizotinib for 10 months. Another follow‐up MRI confirmed disease progression on March 9, 2023, and the patient underwent a second BM resection. The lesion also indicated a deletion in exon 19, MET amplification, and a TP53 mutation. Subsequently, the patient was treated with osimertinib combined with bevacizumab for 3 months. On July 15, 2023, the same lesion recurred (Figure 1B), and a third resection was performed on July 23, 2023. HE and immunohistochemical staining of the final surgical specimen confirmed that the lesion was a proliferative BM tissue originating from NSCLC (Figure 1C).

### 3.2 Establishment and Identification of the AlmoR1 Cell Line

**FIGURE 2:**
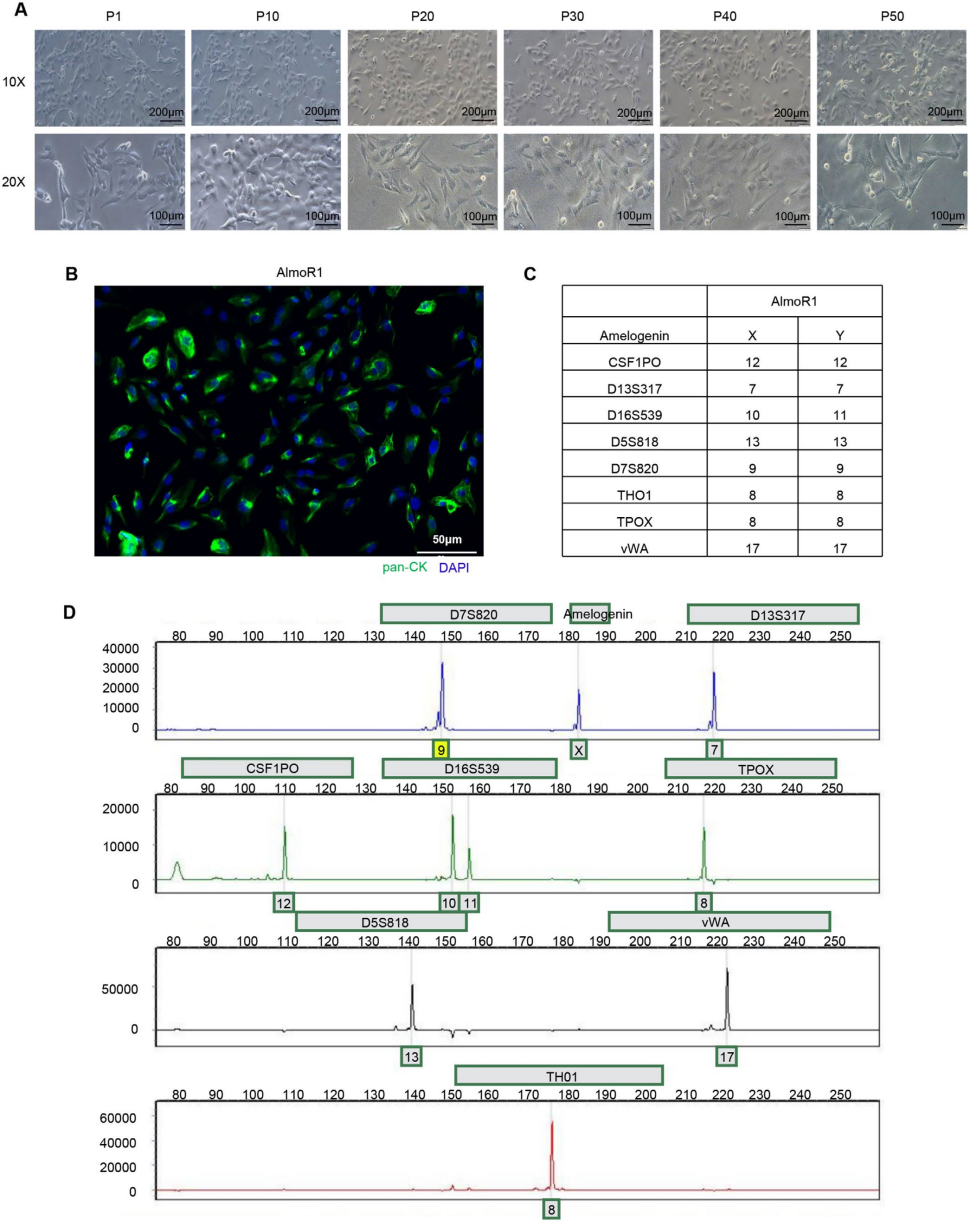
Establishment and identification of the AlmoR1 cell line. (A) Morphology of the AlmoR1 cell line. Inverted photomicrograph of AlmoR1 cells at passages 1, 10, 20, 30, 40, and 50 (10× and 20× magnification, respectively). Scale bar: 100 μm or 200 μm. (B) The expression of pan‐CK in AlmoR1 cells was detected using immunofluorescence technology. The green fluorescence signal represents pan‐CK, while the blue fluorescence signal represents DAPI. Scale bar: 50 μm. (C, D) Unique STR genotype profile of AlmoR1 cell line. An electropherogram of the STR analysis was performed to confirm that the AlmoR1 cell line differed from other known established human cell lines.

We isolated primary cells and purified them using the differential adhesion method from the fresh BM tissue obtained during the final surgery. The cell line named AlmoR1 was established successfully and passaged in vitro consecutively. We periodically observed the morphology of AlmoR1 cells under an inverted microscope during long‐term cultivation in vitro (passage > 50) (Figure 2A). The cells showed a consistent and well‐differentiated morphology. We detected an epithelial marker in AlmoR1 cells by immunofluorescence and found that AlmoR1 cells have a widespread expression of pan‐CK in the cytoplasm, which means AlmoR1 cells were consistent with the resistant BM tissue (Figure 2B). Furthermore, we genotyped the AlmoR1 cell line to exclude the possibility of cross‐contamination with other cell lines. We tested eight STR loci (CSF1PO, D13S317, D16S539, D5S818, D7S820, THO1, TPOX, vWA). AlmoR1 cells had a unique STR profile (Figure 2C,D), indicating that we had established a novel NSCLC BM cell line. The AlmoR1 cell line was stored in the China General Microbiological Culture Collection Center (CGMCC). The deposit number is CGMCC No. 45858.

### 3.3 AlmoR1 Cell Line Exhibits EGFR Exon 19 Deletion Mutation

**FIGURE 3:**
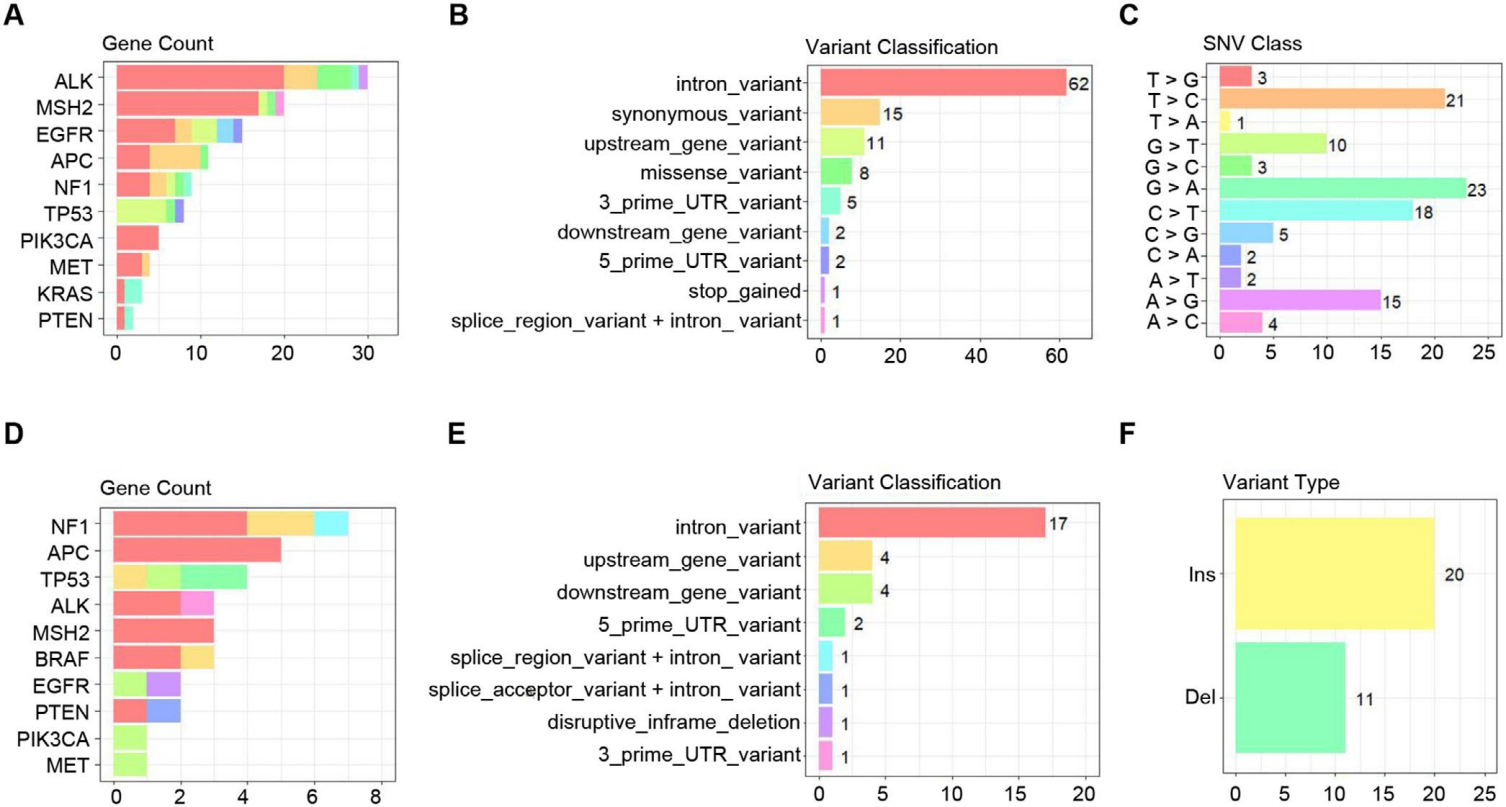
AlmoR1 cell line exhibits EGFR exon 19 deletion mutation. (A) The well‐known lung cancer‐related genes in AlmoR1 cells (TP53, PTEN, PIK3CA, NF1, MSH2, MET, KRAS, EGFR, APC, ALK) exhibit single‐nucleotide polymorphisms (SNPs). (B) The types and frequencies of SNPs in the genes are shown in (A) in AlmoR1 cells. (C) The types and frequencies of base mutations in the SNVs of the genes are shown in (A) in AlmoR1 cells. (D) The well‐known lung cancer–related genes in AlmoR1 cells (TP53, PTEN, PIK3CA, NF1, MSH2, MET, EGFR, APC, ALK, BRAF) exhibit insertion–deletion mutations (InDels). (E) The types and frequencies of InDels in the genes are shown in (D) in AlmoR1 cells. (F) The types and frequencies of base mutations in the InDels of the genes are shown in (D) in AlmoR1 cells.

To elucidate the mutational landscape of the AlmoR1 cell line, we performed WES. Mutations in well‐known lung cancer–related genes (e.g., TP53, PTEN, PIK3CA, NF1, MSH2, MET, KRAS, EGFR, APC, ALK, MET, BRAF) were primarily identified as single‐nucleotide polymorphisms (SNPs) and insertions/deletions (InDels) (Figure 3A–F). The only meaningful alteration was the EGFR E746_A750del mutation, the most common type of exon 19 deletion (Table 1). On the contrary, the ALK and MET mutations were nonsense mutations, as detailed in Tables 2 and 3. These findings indicate that the established lung cancer BM cell line AlmoR1 harbors an EGFR exon 19 deletion mutation.

**TABLE 1:** Specific EGFR mutations in AlmoR1 cells.

Gene	Position	CDS mutation	AA mutation	Type	dbSNP ID
EGFR	chr7:55019087	c.‐191A>C		5_prime_UTR_variant	rs712830
EGFR	chr7:55146655	c.474C>T	p.Asn158Asn	synonymous_variant	rs2072454
EGFR	chr7:55146954	c.*230G>T		downstream_gene_variant	rs2270247
EGFR	chr7:55151210	c.‐1127T>C		upstream_gene_variant	rs2075109
EGFR	chr7:55151466	c.‐871C>T		upstream_gene_variant	rs2075110
EGFR	chr7:55152484	c.629‐62A>G		intron_variant	rs11506105
EGFR	chr7;55,156,097	c.1006+151T>C		intron_variant	rs3735059
EGFR	chr7:55157477	c.*634G>C		downstream_gene_variant	rs4947987
EGFR	chr7:55160360	c.1498+22A>T		intron_variant	rs1558544
EGFR	chr7:55160480	c.1498+142C>T		intron_variant	rs759162
EGFR	chr7;55,165,033	c.1723‐247G>T		intron_variant	rs17336919
EGFR	chr7:55192070	n.‐741A>G		upstream_gene_variant	rs6970262
EGFR	chr7:55198724	c.2709T>C	p.Thr903Thr	synonymous_variant	rs1140475
EGFR	chr7:55200575	c.2946+162C>T		intron_variant	rs139068680
EGFR	chr7:55205133	c.3272‐123G>A		intron_variant	rs2692456
EGFR	chr7:55174771	c.2235_2249delGGAATTAAGAGAAGC	p.Glu746_Ala750del	disruptive_inframe_deletion	rs121913421
EGFR	chr7:55201982	n.*1180_*1181insAG		downstream_gene_variant	rs34723095

Abbreviations: AA mutation: amino acid mutation; CDS mutation: coding sequence mutation; Chr: chromosome ID; dbSNP: single‐nucleotide polymorphism database.

**TABLE 2:** Specific ALK mutations in AlmoR1 cells.

Gene	Position	CDS mutation	AA mutation	Type	dbSNP ID
ALK	chr2:29192926	c.*298C>T		3_prime_UTR_variant	rs1728828
ALK	chr2:29193500	c.4587C>G	p.Asp1529Glu	missense_variant	rs1881421
ALK	chr2:29193615	c.4472A>G	p.Lys1491Arg	missense_variant	rs1881420
ALK	chr2:29193706	c.4381A>G	p.Ile1461Val	missense_variant	rs1670283
ALK	chr2:29194080	c.4165‐158A>G		intron_variant	rs12619135
ALK	chr2:29196547	c.4164+223A>G		intron_variant	rs1728826
ALK	chr2:29196725	c.4164+45C>A		intron_variant	rs1670284
ALK	chr2:29197449	c.4073+93G>A		intron_variant	rs3738870
ALK	chr2:29207022	c.3938+149C>T		intron_variant	rs4666178
ALK	chr2:29207653	c.3837‐381C>A		intron_variant	rs3820712
ALK	chr2:29210116	c.3744‐238G>A		intron_variant	rs6748797
ALK	chr2:29213767	c.3743+217C>A		intron_variant	rs1625283
ALK	chr2:29221210	c.237C>A	p.Phe79Leu	missense_variant	rs1534545
ALK	chr2:29221229	/c.218G>A	p.Trp73*	stop_gained	rs1569156
ALK	chr2:29222592	c.3375C>A	p.Gly1125Gly	synonymous_variant	rs3795850
ALK	chr2:29222932	c.3360‐325A>G		intron_variant	rs4666182
ALK	chr2:29223318	c.3359+24G>C		intron_variant	rs2276550
ALK	chr2:29232150	c.2632+154G>A		intron_variant	rs12714270
ALK	chr2:29232401	c.2535T>C	p.Gly845Gly	synonymous_variant	rs2256740
ALK	chr2:29232872	c.2488‐424C>T		intron_variant	rs4666183
ALK	chr2:29275344	c.1912+58T>C		intron_variant	rs4589708
ALK	chr2:29296756	c.1817+132G>A		intron_variant	rs2272409
ALK	chr2:29297219	c.1648‐162C>T		intron_variant	rs4666194
ALK	chr2:29302475	c.1648‐5418T>C		intron_variant	rs11127213
ALK	chr2:29302479	c.1648‐5422A>G		intron_variant	rs11127214

Abbreviations: AA mutation: amino acid mutation; CDS mutation: coding sequence mutation; Chr: chromosome ID; dbSNP: single‐nucleotide polymorphism database.

**TABLE 3:** Specific MET mutations in AlmoR1 cells.

Gene	Position	CDS mutation	AA mutation	Type	dbSNP ID
MET	chr7:116695959	c.‐14‐3112G>A		intron_variant	rs62469056
MET	chr7:116755203	c.1702‐152C>T		intron_variant	rs38860
MET	chr7:116757518	c.1944A>G	p.Gln648Gln	synonymous_variant	rs13223756
MET	chr7:116781913	c.3577‐75T>C		intron_variant	rs6947629
MET	chr7:116700477	c.*878_*879insT		downstream_gene_variant	rs35212357

Abbreviations: AA mutation: amino acid mutation; CDS mutation: coding sequence mutation; Chr: chromosome ID; dbSNP: single‐nucleotide polymorphism database.

### 3.4 AlmoR1 Cell Line Is Resistant to Third‐Generation EGFR‐TKIs

**FIGURE 4:**
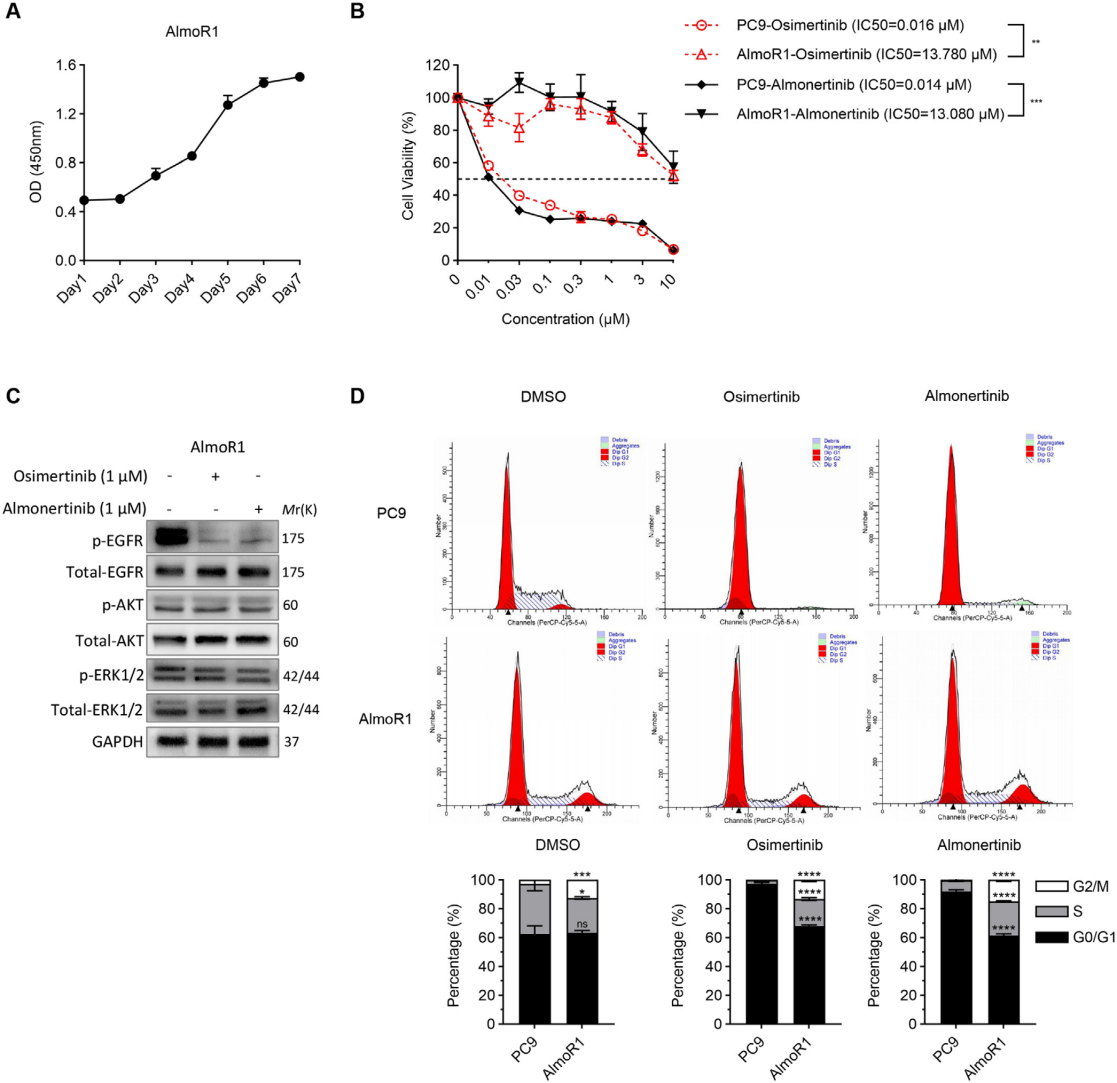
AlmoR1 cell line is resistant to third‐generation EGFR‐TKIs. (A) Continuous monitoring of the proliferation of the AlmoR1 cell line was performed using the CCK8 assay, and a growth curve was plotted. (B) The sensitivity of PC9 and AlmoR1 cells to different concentrations (0, 0.01, 0.03, 0.1, 0.3, 1, 3, and 10 μM) of osimertinib or almonertinib was assessed using the MTT assay. **p < 0.01 and ***p < 0.001. (C) Western blot was used to verify the activation status of EGFR and downstream survival signals AKT and ERK1/2 in AlmoR1 cells in the control group, osimertinib‐treated (1 μM) group, and almonertinib‐treated (1 μM) group. (D) After treatment with 1 μΜ osimertinib or 1 μM almonertinib at indicated concentrations for 72 h, cell cycle distributions of PC9 or AlmoR1 cells were analyzed via flow cytometry. *p < 0.05, **p < 0.01, ***p < 0.001, and ****p < 0.0001.

Using the CCK8 assay, we continuously monitored the proliferation of the AlmoR1 cell line and plotted a growth curve (Figure 4A), which showed an S‐shaped distribution, indicating exponential growth and good condition. To confirm the resistance of AlmoR1 cells to third‐generation EGFR‐TKIs, we employed the MTT assay to assess the sensitivity of EGFR‐mutant PC9 cells and AlmoR1 cells to almonertinib and osimertinib. The IC50 of PC9 to third‐generation EGFR‐TKIs was 0.01 μM, while the IC50 of AlmoR1 was approximately 13 μM, significantly higher than that of sensitive cells (Figure 4B). Furthermore, western blot analysis confirmed that while almonertinib and osimertinib inhibited EGFR phosphorylation, they failed to suppress the activation of downstream survival signals such as AKT and ERK (Figure 4C). Compared to PC9 cells, AlmoR1 cells are more arrested in the G2/M phase, indicating that resistant cells have a more complex cell cycle regulation, which may enhance proliferative capacity by modulating the cell cycle (Figure 4D). These results indicate that the primary lung cancer BM cell line AlmoR1 exhibits widespread resistance to EGFR‐TKIs.

### 3.5 AlmoR1 Cell Line Has Good Intracranial Tumorigenicity

**FIGURE 5:**
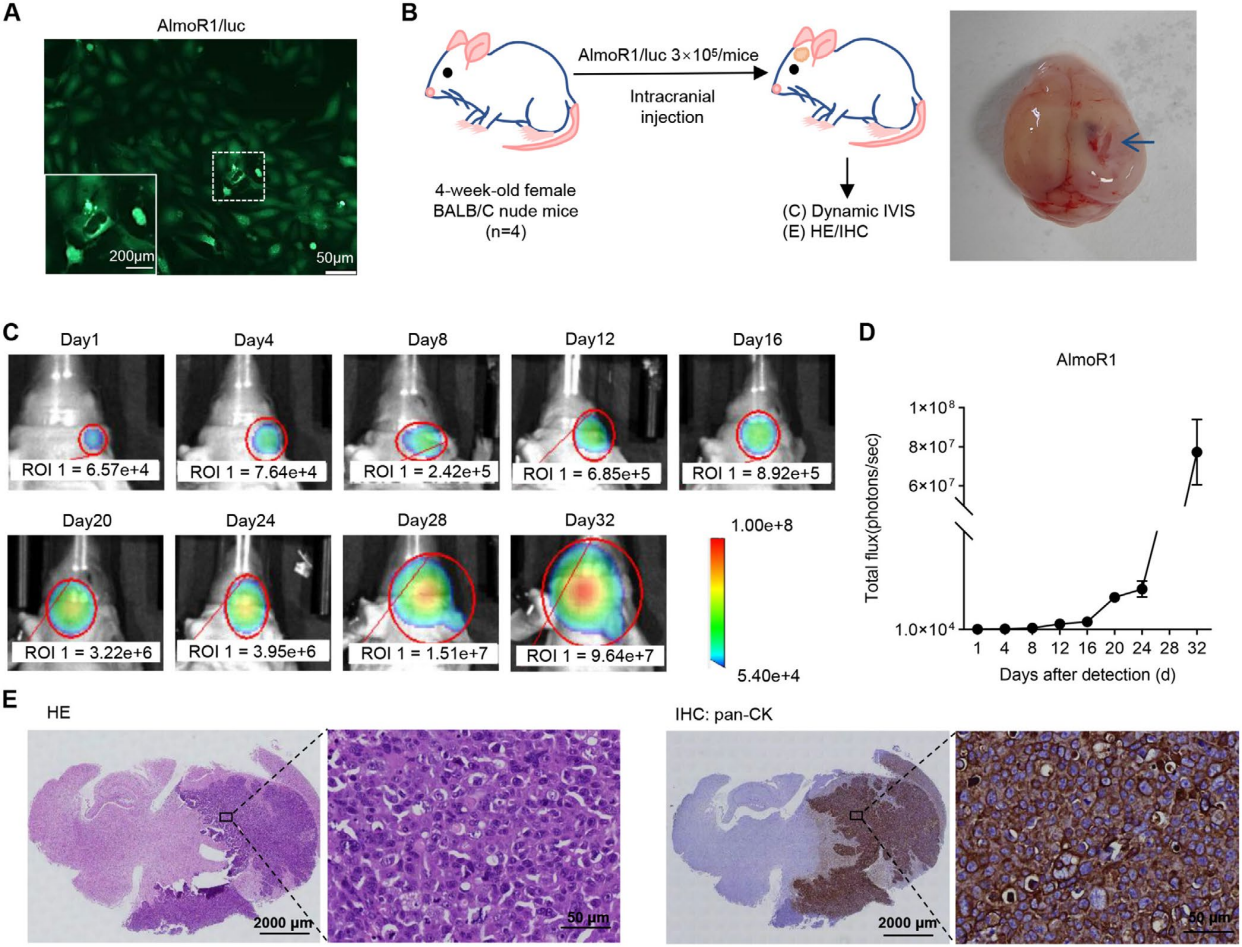
AlmoR1 cell line has good intracranial tumorigenicity. (A) AlmoR1 cells were transduced with a lentivirus carrying the Ubi‐MCS‐frefly_Luciferase‐3FLAG‐CBh‐gcGFP‐IRES‐puromycin (multiplicity of infection = 30) to establish stable luciferase and GFP expression, which was named AlmoR1/luc. (B) Pattern diagram of AlmoR1 cell implanted intracranial tumors in 4‐week‐old female BALB/C nude mice (*n* = 4). Fresh cranial images of AlmoR1 tumor‐bearing nude mice. At the end stage of the disease in the tumor‐bearing BALB/C nude mice, the cranial tissues were extracted. The tumor is indicated by the blue arrow. (C) The representative images of in vivo imaging systems (IVIS) on Days 1, 4, 8, 12, 16, 20, 24, 28, and 32 after the implantation of AlmoR1 cells in nude mouse brain tumors for 3 weeks. (D) The fluorescence curve of AlmoR1 cell orthotopic brain implantation. (E) Representative images of HE staining and pan‐CK immunostaining of brain in situ implantation tumors established by injection of AlmoR1 cells. Scale bar = 2 mm/50 μm.

To evaluate the intracranial tumorigenic potential of AlmoR1 cells, we established an orthotopic brain tumor model for brain tumors using luciferase‐transfected AlmoR1 cells (named AlmoR1/luc) (Figure 5A,B). Approximately 3 weeks after inoculating female BALB/c nude mice with AlmoR1 cells, tumor formation can be observed in the in vivo imaging systems (IVIS), showing a tumorigenic rate of 75% (3/4), and the bioluminescence curve indicates a good state of tumor growth (Figure 5C,D). Subsequently, we extracted the orthotopic brain tumors from mice and performed HE, and pan‐CK, which revealed that the pathological features were highly consistent with those of patient‐derived BM tissues (Figure 5E). Overall, AlmoR1 cells exhibited significant intracranial tumorigenicity.

In conclusion, the AlmoR1 cell line derived from NSCLC BM harbors an EGFR exon 19 deletion mutation and exhibits broad resistance to EGFR‐TKIs. As the first humanized lung cancer BM cell line with a driver gene mutation, it will be a valuable tool for targeted therapy resistance research, particularly in the field of lung cancer BM.

## 4 Discussion

In this study, we successfully established a human‐derived NSCLC brain parenchymal metastasis cell line, AlmoR1. Similar to the well‐established NSCLC BM cell line PC9‐BrM3 (EGFR exon 19 del), this cell line harbors the EGFR E746_A750del (exon 19 del) mutation. However, PC9‐BrM3 remains sensitive to EGFR‐TKIs [26], while AlmoR1 exhibits widespread resistance to third‐generation EGFR‐TKIs. This is the first human‐derived lung cancer brain parenchymal metastasis cell line that both carries a driver gene mutation and demonstrates resistance to third‐generation EGFR‐TKIs.

Current resistance models are primarily either in vitro or in vivo, but in vitro induction of drug resistance removes the cells from their tumor microenvironment [27, 28]. In vivo models include orthotopic BM models, ectopic metastasis models, and intracranial implantation models [29, 30]. However, these models fail to accurately depict the intracranial tumor microenvironment and the process of targeted drug resistance in brain metastases [27, 28]. This significantly hinders the in‐depth investigation of drug resistance mechanisms associated with brain metastases. In contrast, the AlmoR1 cell line, derived directly from a brain parenchymal metastasis, more accurately simulates the real‐world scenario of BM. Furthermore, AlmoR1 exhibits broad resistance to multiple third‐generation EGFR‐TKIs, making it an ideal model for studying resistance mechanisms and screening new drugs targeting NSCLC brain metastases.

Despite the development of acquired resistance to EGFR‐TKIs, both pre‐ and posttreatment BM specimens retained the EGFR exon 19 deletion mutation. Given that EGFR signaling in AlmoR1 cells was inhibited by osimertinib and almonertinib, yet downstream survival pathways such as AKT and ERK remained persistently activated, we hypothesize that acquired resistance to EGFR‐TKIs may not be solely driven by the EGFR mutation itself but rather involve alternative compensatory survival pathways that regulate cell cycle progression and cell survival [17, 31]. In this study, AlmoR1 cells maintained their original morphology, and histological analysis (HE staining) of AlmoR1‐derived tumor tissues from in vivo models showed consistency with the patient's BM specimens. These suggests that small cell transformation is unlikely to be the underlying resistance mechanism. To explore the possibility of bypass signaling activation, we examined several well‐known pathways, including MET amplification and ALK rearrangement, but none were detected. Thus, the precise mechanism underlying AlmoR1's resistance to third‐generation EGFR‐TKIs remains unclear and requires further investigation in future studies.

In summary, the establishment of the AlmoR1 cell line is important for NSCLC BM research. This cell line provides a unique model that better aligns with clinical settings and offers distinct advantages in investigating targeted resistance mechanisms in EGFR‐mutated lung cancer brain metastases.

## Author Contributions

**Jingyi Wu:** data curation (equal), validation (equal), writing – original draft (equal). **Xiangfei Wu:** investigation (equal), writing – original draft (equal). **Jing Wang:** formal analysis (equal). **Guili Feng:** methodology (equal), resources (equal). **Yuhan Wang:** validation (equal). **Zide Chen:** software (equal), visualization (equal). **Wei Wang:** supervision (equal), writing – review and editing (equal). **Rong Wang:** conceptualization (equal), funding acquisition (equal), project administration (equal), supervision (equal), writing – review and editing (equal).

## Ethics Statement

Approval of the research protocol was obtained by an institutional reviewer board. This study was approved by the Ethics Committee of Guangdong Sanjiu Brain Hospital (Affiliated Brain Hospital of Jinan University) (Ethical No. 2024‐02‐064). All informed consent was obtained from the subject and guardians. Animal Studies: All animal experiments were conducted in accordance with the ethical guidelines of the Animal Experimentation Committee of Nanfang Hospital (Guangzhou, China) (Approval No. IACUC‐LAC‐20230519‐001).

## Conflicts of Interest

The authors declare no conflicts of interest.

## Data Availability

The authors have nothing to report.
